# WHICH CAME FIRST, WELL-BEING OR PHYSICAL HEALTH? A LONGITUDINAL INVESTIGATION IN MID- TO LATE LIFE

**DOI:** 10.1093/geroni/igz038.2642

**Published:** 2019-11-08

**Authors:** Tarah Raldiris, Joseph Dzierzewski

**Affiliations:** Virginia Commonwealth University, Richmond, Virginia, United States

## Abstract

As the United States population continues to age, focus has turned toward understanding and promoting positive aging processes. However, positive aging is not only about maintaining physical health, but also about maintaining and improving psychological health. Though previous research has found well-being to be predictive of physical health outcomes, research has yet to examine the temporal associations between these variables. The aim of the current study was to begin to disentangle these temporal associations by investigating how well-being and physical health relate over the course of nearly two decades in a nationwide sample of adults (N = 7,419, Mage = 46.38 at Time 1). The current study employed a cross-lagged panel SEM design across three time points to investigate if well-being, operationalized as purpose in life and personal growth, predicted future self-rated physical health. Data from the Midlife in the United States (MIDUS) study was analyzed from 1995, 2004, and 2014. Cross-lagged analyses revealed well-being was significantly predictive of future physical health, and physical health was also significantly predictive of future well-being. However, the magnitude of the path loadings indicated well-being was a stronger predictor of future physical health than physical health was of future well-being. Thus, these results suggest that personal growth and purpose in life may be particularly important for the promotion of physical health as individuals age. Future research should investigate if these predictive associations apply to all age-groups, as well as for individuals who self-identify as caregivers.

